# Ecological effects of cefepime use during antibiotic cycling on the Gram-negative enteric flora of ICU patients

**DOI:** 10.1186/s40635-018-0185-2

**Published:** 2018-07-27

**Authors:** Carola Venturini, Andrew N. Ginn, Brooke E. Wilson, Guy Tsafnat, Ian Paulsen, Sally R. Partridge, Jonathan R. Iredell

**Affiliations:** 1 Centre for Infectious Diseases and Microbiology, The Westmead Institute for Medical Research, The University of Sydney and Westmead Hospital, Sydney, NSW Australia; 2 Australian Institute of Health Innovation, Centre for Health Informatics, Macquarie University, Sydney, NSW Australia; 3 Spokade Pty Ltd., Sydney, Australia; 4 Faculty of Science and Engineering, Department of Chemistry and Biomolecular Sciences, Macquarie University, Sydney, NSW Australia

## Abstract

This study examines the impact of cefepime and APP-β (antipseudomonal penicillin/ β-lactamase inhibitor combinations) on Gram-negative bacterial colonization and resistance in two Australian ICUs. While resistance did not cumulatively increase, cefepime (but not APP-β treatment) was associated with acquisition of antibiotic resistant Enterobacteriaceae, consistent with an ecological effect. Analysis of the resident gut E. coli population in a subset of patients showed an increase in markers of horizontal gene transfer after cefepime exposure that helps explain the increase in APP-β resistance and reminds us that unmeasured impacts on the microbiome are key outcome determinants that need to be fully explored.

**Electronic supplementary material:**

The online version of this article (10.1186/s40635-018-0185-2) contains supplementary material, which is available to authorized users.

To the Editor,

Effects of late-generation cephalosporins such as cefepime (FEP) on resistance acquisition and the gut microflora are uncertain [1–4]. In a previous study in two Australian ICUs, nearly 70% of all prescriptions were allocated in respective cycles to either cefepime or an antipseudomonal penicillin/β-lactamase inhibitor (APP-β) like piperacillin/tazobactam [5]. Under this strong sustained selection, cefepime exposure resulted in more methicillin-resistant *Staphylococcus aureus* (MRSA) and *Pseudomonas aeruginosa* colonization and infection than APP-β despite equivalent in vitro susceptibility [5]. In order to determine whether clinically important Enterobacteriaceae were similarly affected, perineal samples from patients within this cohort who had been admitted directly to the ICU (*n* = 206) were cultured at admission (< 48-h ICU stay) and again after 3 days of cycle-specified antibiotic (FEP or APP-β) [5]. Resistance to gentamicin and APP-β were chosen as key phenotypes not associated with cefepime resistance. Resistant Enterobacteriaceae were cultured from a modest proportion at admission (to gentamicin, 14%; to APP-β, 26%) but with no cumulative increase over time, as for MRSA and *Pseudomonas* [5]. Colonization by APP-β-resistant Enterobacteriaceae increased significantly overall after ICU admission (*p* = 0.015) but was almost 2.5 times more likely after cefepime than APP-β exposure (*p* < 0.05) (Fig. 1).

**Fig. 1:**
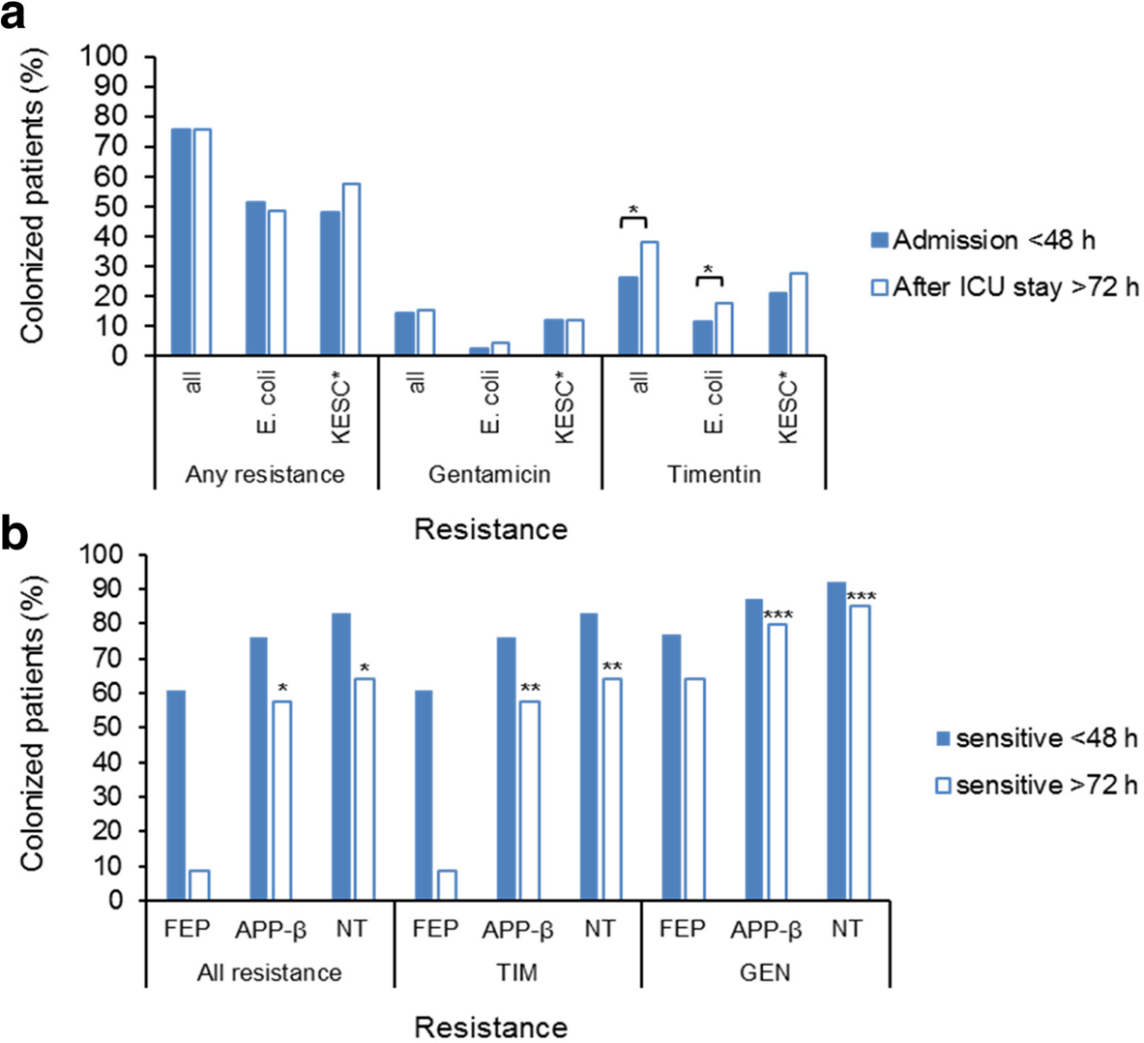
Variation in colonization patterns before and after ICU stay. **a** Rates of colonization with antibiotic-resistant species at admission and after ICU stay. **b** Change in susceptibility rates after ICU stay in different treatment cycles. Proportion of the entire study group of patients (*n* = 206) with positive cultures. “Any resistance” indicates any bacterial growth on antibiotic supplemented media. “All resistance” indicates any growth on ChromAgar™ supplemented with vancomycin only (to exclude Gram-positive species). *KESC, *Klebsiella*, *Enterobacter*, *Serratia*, *Citrobacter* spp. as identified on colorimetric media ChromAgar™ and confirmed by MALDI-TOF [16] as previously described [5]. Asterisks (*, **, ***) above bar charts indicate significant differences (*p* < 0.05)

Patients without resistant Enterobacteriaceae on admission were more likely to remain free of them after treatment with APP-β (or no drug) than cefepime (Fig. 1; *p = 0.004), and this association held when APP-β and cefepime treatment were directly compared (*n* = 45 vs. 22, *p* = 0.016). This was also true for APP-β (Fig. 1; **p = 0.002) and gentamicin resistance (Fig. 1; ***p = 0.014) when considered individually (Table 1). Backwards stepwise logistic regression analysis also linked cefepime exposure more strongly to later APP-β-resistance (OR 2.285 (CI 1.096–4.764); *p* = 0.027) than length of stay, age, or admission APACHE II score. Cefepime was also more strongly associated with APP-β-resistant *Escherichia coli* than APP-β itself (Pearson’s chi-square test, *p* = 0.027).

**Table 1:** Effect of antibiotic on gain and loss of resistance in Enterobacteriaceae after 72 h in ICU

Resistance	Treatment^a^	Gained^b^	Lost^b^	No change^c^	
				Sensitive	Resistant
Timentin and/or gentamicin	Cefepime	15	7	22	17
	APP-β	14	6	45^c^	13
	None	13	3	43^a^	8
		*p* = 0.610	*p* = 0.34	*p* = 0.004^a^	*p* = 0.07
Timentin	Cefepime	15	8	22	16
	APP-β	14	6	45^a^	13
	None	12	3	44^a^	8
		*p* = 0.550	*p* = 0.20	*p* = 0.002^a^	*p* = 0.10
Gentamicin	Cefepime	8	7	39	7
	APP-β	6	8	62^a^	2
	None	5	2	57^a^	3
		*p* = 0.456	*p* = 0.16	*p* = 0.014^a^	*p* = 0.06

^a^APP-β, antipseudomonal penicillin/β-lactamase; none, no cefepime or APP-β

^b^Number of patients

^c^Significant difference (*p* < 0.05)

Our analysis showed that high-level homogeneity of β-lactam antibiotics within cycles was not associated with overall increased resistance, in agreement with other studies on antibiotic cycling in which Gram-negative bacterial susceptibility was not significantly altered [2, 6, 7]. The apparent ecological effects that we describe are consistent with our own data regarding MRSA and *P*. *aeruginosa* [5], challenging antimicrobial homogeneity as a driver of resistance per se [8], an idea that was premised on a mathematical model which was recently disputed [9]. Antibiotic use is recognized as the single most powerful selective pressure for the emergence of resistance particularly in environments where usage is high (ICU). However, the different strategies implemented to curb the rise of resistance in hospitals, including cycling, have had variable outcomes due to the complex relationship between use of specific drugs and resistance patterns in bacterial populations [10]. In our study, despite stable overall resistance rates, treatment with cefepime was a significant independent predictor of acquisition of antibiotic-resistant Gram-negative organisms and was also strongly associated with increased resistance to APP-β, but not to cefepime or extended-spectrum β-lactams (Table 1), in agreement with other studies on cefepime use in hospitalized patients [6, 11].

Our data strongly point to in vivo ecological effects of antibiotics rather than specific selection pressure associated with use of a specific antimicrobial class. However, ecological perturbation does not readily explain gentamicin and β-lactam resistance after cefepime, as these phenotypes are typically plasmid-encoded in the Enterobacteriaceae. We therefore directly compared *E*. *coli* populations from each of 12 patients before and after cefepime treatment (Additional file 1: Methods) and found no increase in virulence-associated types nor dominance of any single resistant clone (Table 2). Cultured isolates were of limited diversity, almost all of the B2 and D phylogenetic subtypes. There were three or less clearly distinguishable restriction types, and antibiotic resistance phenotypes gave no hint of underlying processes. However, a general effect on mobile genetic elements was suggested by the increased complexity and abundance of self-transmissible resistance plasmids and by enrichment for mobile resistance genes not relevant to cefepime (e.g., *strAB*, *bla*_TEM_, *bla*_SHV_; Fig. 2).

**Table 2:** Antimicrobial resistance (AR) profiles of isolated *E*. *coli* representatives

Patient	Isolate^†^	AR phenotype^‡^	AR genotype^§^
1B	a B2 a D/E	None None	None
1A	c B2	None	None
2B	d B2 e B2	None None	None
2A	e B2 d B2	None None	None
3B	f B2 f_1_ B2 h D/E	None TET TET	*tet*(B) *aphA1 dfrA14 strA strB sul2*
3A	f_1_ B2-D/E h D/E	TET TET	*tet*(B)
4B	i B2	AMP AMC CFZ*i* TIM*i*	*bla*_TEM_ *sul2*
4A	k B2 m D/E	AMP CFZi CHL*i* AMP	*aadA bla*_SHV_ In
5B	n B2	AMP CFZ*i* TIM*i*	*aadA bla*_TEM_ In
5A	n B2	AMP CFZ*i* TIM*i*	*aadA bla*_TEM_ In
6B	p B2-unk	AMP AMC TZP TIM CHL	*aadA bla*_OXA-1_ *catA1* In
6A	q B2 r B2	None AMP CFZ*i* TIM*i* TET	*bla*_TEM_ *tet*(A)
7B	s F	AMP CFZ*i* TMP SXT	*bla*_TEM_ *dfrA14 sul2* *strA strB*
7A	s_1_ D/E s_2_ F s_2_ D/E	AMP AMC*i* CFZ TZP TIM TMP SXT AMP AMC*i* CFZ TZP TIM TOB*i* TMP SXT AMP AMC*i* CFZ TIM TMP SXT	*bla*_TEM_ *dfrA14* *strA strB* *sul2*
8B	t B2	AMP CFZ*i* TIM*i*	*aadA* *bla*_TEM_ In
8A	t B2	AMP AMC*i* CFZ TIM	*aadA* *bla*_TEM_ In
9B	u B2 v B2	AMP AMC*i* CFZ*i* None	*bla*_TEM_
9A	u B2	AMP AMC*i* CFZ*i* TIM*i*	*bla*_TEM_
10B	z B1 z_1_ B1 z_2_ B1	TET TET TET	*tet*(B)
10A	w B2 y unk	None AMP TIM*i* TMP SXT	*bla*_TEM_ *dfrA5* *strA strB* *sul2*
11B	w B2	None	none
11A	aa D/E	AMP AMC AZ CFZ FOX CAZ CRO LEX TIM*i*	*bla*_CMY-2_-like
12B	bb B1 cc B1	AMP CFZ*i* TIM*i* TMP SXT AMP TIM*i* CHL*i* TMP SXT	*bla*_TEM_ *catA1 dfrA7* In
12A	dd B2 ee B2	AMP AMC*i* CFZ*i* TIM AMP AMC*i* CFZ TIM*i*	*bla*_TEM_

Underlined data not detected phenotypically by the BD Phoenix^TMP^ system

B before antibiotic treatment (< 48 h ICU stay), A after antibiotic treatment (≥ 72 h ICU stay), *i* intermediate, In class 1 integron 5′- and/or 3′-conserved segments

^†^Defined by PFGE pattern (“a” to “ee”) and by phylogenetic grouping (A, B1, B2, D/E, F, unk (unknown) [19])

^‡^Not susceptible by BD Phoenix^TMP^ screening of single *E*. *coli* colonies

^§^Genotype determined by NGS sequencing data analysis of pooled *E*. *coli* representatives for each patient, using BLAST comparisons [20] to the MARA database [17] and our in-house database of *rep* and mobilization genes (Additional file 1: Table S1)

**Fig. 2:**
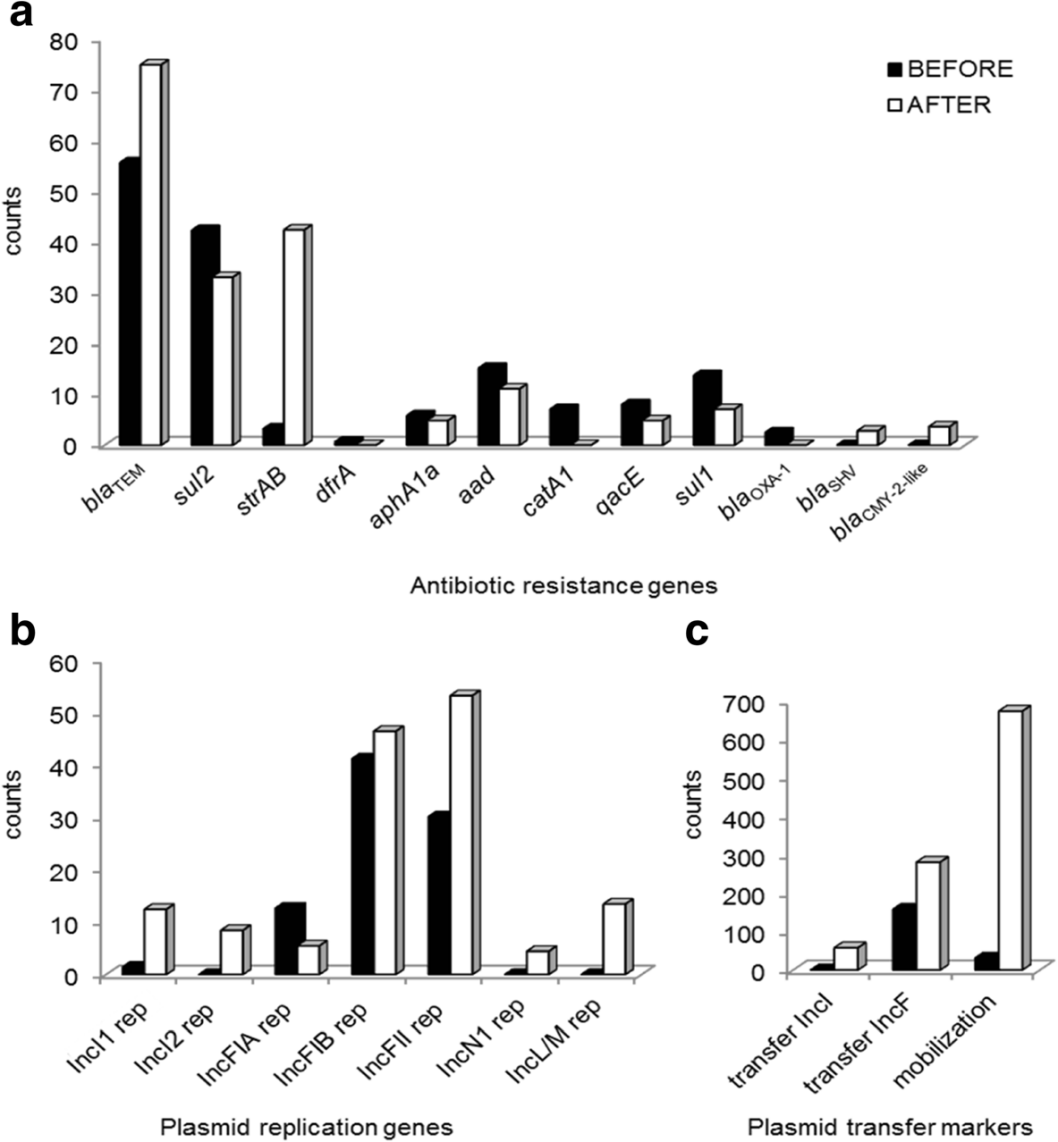
Antibiotic resistance markers detected before and after cefepime exposure in *E*. *coli* isolates from ICU patients. Resistance genes (**a**), plasmid replication genes (**b**), and plasmid transfer markers (**c**) were identified using the sequencing output from MiSeq sequencing (250 bp; paired-end) of pooled representatives (2–6 colonies per patient) of the *E*. *coli* population before (black) and after (white) cefepime treatment. Markers were detected by alignment with resistance genes and mobile genetic elements in the MARA database [17] and plasmids markers in our in-house database (Additional file 1 Methods). *aad* includes *aadA1*, *aadA6*, and *aadA10*; “transfer” comprises marker genes for conjugation/self-transmission (*tra*, *nik*, etc.); “mobilization” indicates relaxase genes of mobilizable plasmid types (Additional file 1: Table S1) [18]

In animal models, a proteobacterial bloom that accompanies colitis was associated with accelerated plasmid transfer between species [12], and a similar proteobacterial bloom is relatively prolonged after third-generation cephalosporins compared to penicillins [13, 14], providing a potential biological explanation for our findings (Fig. 2). Antibiotic treatment modifies the microbial community structure in the gut by shifting the competitive balance between sensitive bacteria and resistant/pathogenic subpopulations [15]. These subpopulations carry different resistant determinants that may come to predominate both by amplification of the original carriers and/or spread to other species. In Gram-negative enterobacteria, antibiotic resistance develops mainly via horizontal transfer of resistance genes that often cluster together in the same genetic locus, either on the chromosome or on plasmids, giving rise to multiple resistant types. Use of one antibiotic may drive selection of resistance to an entirely different class of drugs due to both cross-resistance mechanisms and co-localization of genetic elements. Perhaps more importantly resistance determinants are also associated with diverse mobile genetic elements (transposons, insertion sequences, plasmids) that allow for the movement of multidrug resistance loci between bacterial cells [15].

Even though selection and spread of specific resistance might be constrained by fitness requirements, antibiotic activity itself is known to promote horizontal gene transfer by triggering recombination and conjugation events, which will affect population-level resistance patterns [15], and by acceleration of gene transfer during population expansion events [13]. Together, these data indicate that cefepime exposure differentially drives antibiotic resistance in the microflora other than by direct phenotypic selection and are consistent with descriptions of enhanced plasmid transfer in other gut dysbioses [13]. This provides a potential explanation for resistance (e.g., to extended-spectrum β-lactam antibiotics) in Enterobacteriaceae that has been linked to exposure to late-generation cephalosporins, such as cefepime [14], and seems likely generalizable to third-generation cephalosporins, which have similar activities, gut penetration and associations with antibiotic resistance. It appears unlikely from (narrower-spectrum) first-generation cephalosporins, but reminds us that unmeasured impacts on the microbiome are key outcome determinants that have yet to be fully explored.

## Additional file

Additional file 1: Methods. This file describes the methods used to obtain and analyze the data presented in this manuscript and includes **Table S1.** (entitled “Markers for transmissible antibiotic resistance included in our in-house screening”) and additional references pertaining to the methodology. (DOCX 24 kb)
