# Supplementary material for: Ecological effects of cefepime use during antibiotic cycling on the Gram-negative enteric flora of ICU patients
Source: Intensive Care Med Exp. 2018 Jul 27;6:19. doi: 10.1186/s40635-018-0185-2 (PMC6063807; doi:10.1186/s40635-018-0185-2)
Supplement: Supplementary file 1 — Methods. This file describes the methods used to obtain and analyze the data presented in this manuscript and includes Table S1. (entitled “Markers for transmissible antibiotic resistance included in our in-house screening”) and additional references pertaining to the methodology. (DOCX 24 kb) [file 40635_2018_185_MOESM1_ESM.docx]

**Additional file 1: Methods**

Our previous cycling study was designed as a FEP/APP-β repeated schedule of 4-month cycles over 16 months [S1]. In the present sub-study, we analyzed data from a subset of patients (n = 206) admitted to ICU directly from the community with complete microbiological sampling (perineal swabs and/or endotracheal aspirates) collected both at < 48 h from admission (community-acquired) and after > 72 h ICU stay. We selected only patients treated with the preferred antibiotic within a cycle and only cycles with overall high compliance [S1]. We also included bacterial isolates from a control group who received no relevant antibiotics (NT; control).

Identification of isolated Enterobacteriaceae was performed as previously described [S1]. Statistical analysis was performed using SPSS v22 (IBM Corp). Proportions were compared using the Chi-Squared test or Mann-Whitney U. Analysis of multiple predictors of colonization and resistance rates was performed using step-wise backwards logistic regression models, considering only those factors significant at p ≤ 0.10 in univariate analysis, and retaining only factors significant at p ≤ 0.05 in the final model.

**Microbial profiling**

Enterobacteriaceae were isolated and identified as described [S1]. Resistance rates were determined by growth on selective agar (ticarcillin/clavulanate (TIM) (APP-β), 128/2 μg/mL; cefepime (FEP), 64 μg/mL; gentamicin (GEN), 10 μg/mL) and using the BD Phoenix automated system (Becton Dickinson, Sparks, MD, USA) with minimal inhibitory concentration (MIC) breakpoints as outlined by the European Committee on Antimicrobial Susceptibility Testing 2017 guidelines (EUCAST, v 7.1, 2017; http://www.eucast.org/clinical_breakpoints).

For more detailed analysis of FEP effects, single *E. coli* colonies were randomly selected from a subset of 12 patients before (B = ≤ 48 h post ICU admission; n ≤ 6) and after (A = ≥ 72 h; n ≤ 6) FEP treatment and subjected to pulsed-field gel electrophoresis (PFGE) after overnight digestion with *Xba*I (New England Biolabs, Ipswich, MA) [S2]. To discriminate virulence types, *E. coli* phylogenetic groups (A, B1, B2, D/E, F) were assigned as previously described [S3].

**Sequencing**

Equal volumes of genomic DNA extracted from each *E. coli* colony (n ≤ 6) (DNeasy Blood and Tissue Kit, Qiagen, Hilden, Germany) were pooled for each patient. Illumina MiSeq paired-end (250 bp) DNA library preparations were pooled for sequencing and read quality was verified using FastQC (Babraham Bioinformatics). Bioinformatic analysis of raw reads to identify genetic elements associated with antibiotic resistance was performed using Geneious v9.1.2 (Biomatters Ltd) by BLAST-based [S4] comparisons the MARA database [S5] (transmissible antibiotic resistance genes and associated elements (5'- and 3'-conserved segments of class 1 integrons), plasmid replicase (*rep*) and relaxase (*mob*/*traI*/*nikB*) genes) (Supplementary Table 1) [S6].

**Additional file 1: Table S1 Markers for transmissible antibiotic resistance included in our in-house screening.**

| **Name** | **Accession No. (NCBI)** | **Function** |
| --- | --- | --- |
| IncI1 *rep* | AP005147 | plasmid replication |
| IncI2 *rep* | AP002527 | plasmid replication |
| IncFIA *rep* | AP001918 | plasmid replication |
| IncFIB *rep* | AP001918 | plasmid replication |
| IncFII *rep* | AP000342 | plasmid replication |
| IncN1 *rep* | AY046276 | plasmid replication |
| IncL/M *rep* | AF550415 | plasmid replication |
| IncZ *rep* | CU928147 | plasmid replication |
| IncI1 *nikB* | AP005147 | plasmid transfer |
| IncI2 *nikB* | AP002527 | plasmid transfer |
| IncFII *traI* | AP000342 | plasmid transfer |
| pColK-K235 *mbkA* | AY929248 | plasmid mobilization |
| pO26_3 *mbeA* | AP010956 | plasmid mobilization |
| p12579_3 *mobA* | CP003112 | plasmid mobilization |
| ColE1 *mbeA* | J01566 | plasmid mobilization |
| IncQ1 *mobA* | M28829 | plasmid mobilization |

**Additional References**

S1. Ginn AN, Wiklendt AM, Gidding HF, George N, O’Driscoll JS, Partridge SR, O’Toole BI, Perri RA, Faoagali J, Gallagher JE, Lipman J, Iredell JR (2012) The ecology of antibiotic use in the ICU: homogeneous prescribing of cefepime but not Tazocin selects for antibiotic resistant infection. PLoS ONE 7(6):e38719.

S2. Agyekum A, Fajardo-Lubian A, Ansong D, Partridge SR, Agbenyega T, Iredell JR (2016) bla_CTX-M-15_ carried by IncF-type plasmids is the dominant ESBL gene in Escherichia coli and Klebsiella pneumoniae at a hospital in Ghana. Diagn Microbiol Infect Dis 84(4):328–333.

S3. Clermont O, Christenson JK, Denamur E, Gordon DM (2013) The Clermont *Escherichia coli* phylo-typing method revisited: improvement of specificity and detection of new phylo-groups. Environ Microbiol *Rep* 5(1):58–65.

S4. Altschul SF, Gish W, Miller W, Myers EW, Lipman DJ (1990) Basic local alignment search tool. J Mol Biol 215(3):403–410.

S5. Partridge SR, Tsafnat G (2018) Automated annotation of mobile antibiotic resistance in Gram-negative bacteria: the Multiple Antibiotic Resistance Annotator (MARA) and database. J Antimicrob Chemother doi:10.1093/jac/dkx513.

S6. Garcillán-Barcia MP, Alvarado A, de la Cruz F (2011) Identification of bacterial plasmids based on mobility and plasmid population biology. FEMS Microbiol Rev 35(5):936–956.
